# Transcriptomic analysis of grain amaranth (*Amaranthus hypochondriacus*) using 454 pyrosequencing: comparison with *A. tuberculatus*, expression profiling in stems and in response to biotic and abiotic stress

**DOI:** 10.1186/1471-2164-12-363

**Published:** 2011-07-13

**Authors:** John P Délano-Frier, Hamlet Avilés-Arnaut, Kena Casarrubias-Castillo, Gabriela Casique-Arroyo, Paula A Castrillón-Arbeláez, Luis Herrera-Estrella, Julio Massange-Sánchez, Norma A Martínez-Gallardo, Fannie I Parra-Cota, Erandi Vargas-Ortiz, María G Estrada-Hernández

**Affiliations:** 1 Unidad de Biotecnología e Ingeniería Genética de Plantas, (Cinvestav-Unidad Irapuato) Km 9.6 del Libramiento Norte Carretera Irapuato-León. Apartado Postal 629, C.P. 36821, Irapuato, Gto., México; 2 Laboratorio Nacional de Génomica para la Biodiversidad, Km 9.6 del Libramiento Norte Carretera Irapuato-León. Apartado Postal 629, C.P. 36821, Irapuato, Gto., México; 3 Department of Entomology, College of Agricultural Sciences. Penn State University, University Park, PA 16802, USA

## Abstract

**Background:**

*Amaranthus hypochondriacus*, a grain amaranth, is a C4 plant noted by its ability to tolerate stressful conditions and produce highly nutritious seeds. These possess an optimal amino acid balance and constitute a rich source of health-promoting peptides. Although several recent studies, mostly involving subtractive hybridization strategies, have contributed to increase the relatively low number of grain amaranth expressed sequence tags (ESTs), transcriptomic information of this species remains limited, particularly regarding tissue-specific and biotic stress-related genes. Thus, a large scale transcriptome analysis was performed to generate stem- and (a)biotic stress-responsive gene expression profiles in grain amaranth.

**Results:**

A total of 2,700,168 raw reads were obtained from six 454 pyrosequencing runs, which were assembled into 21,207 high quality sequences (20,408 isotigs + 799 contigs). The average sequence length was 1,064 bp and 930 bp for isotigs and contigs, respectively. Only 5,113 singletons were recovered after quality control. Contigs/isotigs were further incorporated into 15,667 isogroups. All unique sequences were queried against the nr, TAIR, UniRef100, UniRef50 and Amaranthaceae EST databases for annotation. Functional GO annotation was performed with all contigs/isotigs that produced significant hits with the TAIR database. Only 8,260 sequences were found to be homologous when the transcriptomes of *A. tuberculatus *and *A. hypochondriacus *were compared, most of which were associated with basic house-keeping processes. Digital expression analysis identified 1,971 differentially expressed genes in response to at least one of four stress treatments tested. These included several multiple-stress-inducible genes that could represent potential candidates for use in the engineering of stress-resistant plants. The transcriptomic data generated from pigmented stems shared similarity with findings reported in developing stems of Arabidopsis and black cottonwood (*Populus trichocarpa*).

**Conclusions:**

This study represents the first large-scale transcriptomic analysis of *A. hypochondriacus*, considered to be a highly nutritious and stress-tolerant crop. Numerous genes were found to be induced in response to (a)biotic stress, many of which could further the understanding of the mechanisms that contribute to multiple stress-resistance in plants, a trait that has potential biotechnological applications in agriculture.

## Background

The genus *Amaranthus *L. (Caryophyllales: Amaranthaceae) comprises C4 dicotyledonous herbaceous plants classified into approximately 70 species. It has a worldwide distribution, although most species are found in the warm temperate and tropical regions of the world [1,2]. Many amaranth species are cultivated as ornamentals or a source of highly nutritious pseudocereals (e.g. *grain amaranths*) and vegetables; others, are notoriously aggressive weeds that affect many agricultural areas of the world [3,4]. The grain amaranths (predominantly *Amaranthus hypochondriacus *L., *A. cruentus *L., and *A. caudatus *L.) are ancestral crops native to the New World. They are classified along with their putative progenitor species (*A. hybridus *L., *A. quitensis *H.B.K., and *A. powellii *S. Wats.) in what is known as the *A. hybridus *complex [5]. Restricted for centuries to a limited cultivation in Meso America as a result of religious intolerance [6], grain amaranths have gradually acquired renewed interest due to their various nutritional [7-12] and health-related traits [13], in addition to their highly desirable agronomic characteristics. These characteristics offer a viable alternative to cereals and other crops in many stressful agricultural settings, particularly those where soil moisture conditions vary considerably between growing seasons [14-16]. The increased ability to withstand drought stress that characterizes grain amaranth is closely related to its superior water use efficiency (WUE) [17-20], variously defined as the ratio of economic yield to evapo-transpiration or of the amount CO_2 _assimilated to water loss [21,22]. WUE in grain amaranth has been found to be higher than in other C3 and C4 crops, including wheat, corn, cotton and sorghum [23]. Moreover, the high salt tolerance of grain amaranth has also been associated with a high WUE [16]. The drought-tolerance of grain amaranth has been attributed to the inherently stress-attenuating physiology of the C4 pathway, an indeterminate flowering habit and the capacity to grow long taproots and develop an extensive lateral root system in response to water shortage in the soil [20,24,25]. Recently, the results of a combined proteomic/genomic approach suggested that amaranth's root response to drought stress involves a coordinated response that includes osmolyte accumulation and the activation of stress-related genes needed for the scavenging of reactive oxygen species, protein stabilization and transcriptional regulation of plant growth [26,27].

The use of molecular tools for the advanced genomic study of the genus *Amaranthus *has recently increased, with at least six published reports appearing in the last three years. The construction of a bacterial artificial chromosome (BAC) library for *A. hypochondiacus *representing a 10.6-X coverage of its haploid genome content was reported in 2008 [28]. Shortly afterwards, this BAC library was utilized to generate a set of microsatellite markers for the grain amaranths, which were used to clarify taxonomic relationships within the *A. hybridus *complex. Additional applicability for these microsatellite markers for the study of other economically important species within the *Amaranthus *genus, including weeds and ornamentals, was proposed [29,30]. The utilization of next-generation 454 pyrosequencing technology was subsequently explored as a tool to obtain genomic data for waterhemp (*A. tuberculatus*), a notorious weed of maize and soybean crops in the USA [31]. The sequence data obtained (43 Mbp), which covered 10% of this species' genome, included the nearly complete sequence of the chloroplast genome and revealed genomic data pertaining herbicide resistance genes, simple sequence repeat markers, and repeated elements (e.g., transposons). This materialized later with the publication of a deep coverage of waterhemp's transcriptome that yielded a total of 44,469 unigenes, 49% of which displayed highly significant similarities to *Arabidopsis *proteins [32]. Moreover, this study generated preliminary sequence information for all of the major herbicide target-site genes for which waterhemp has documented resistance, in addition to two other herbicide targets not previously reported as having evolved resistance in any plant species. Similarly impressive results were obtained when more than 500 Mbp sequence data, derived from a single 454-pyrosequencing run, were utilized in combination with novel genomic reduction protocol to discover thousands of single nucleotide polymorphisms in different populations of *A. caudatus *[33].

The information regarding resistance responses to insects and pathogens in amaranth is relatively scarce. The limited number of defense-related genes reported includes protease and α-amylase inhibitors, agglutinins, anti-microbial peptides and ribosome-inactivating proteins [34-39]. This information, however, was complemented by a recent study describing several more insect- and pathogen-induced genes [40]. Similarly limited is the genetic information underlying the mechanisms that confer amaranth with its capacity to withstand drought and/or saline stress, although several abiotic-stress-related genes have been identified in amaranth and in phylogenetically related species such as spinach, cultivated and wild species of beet root, *Mesembryanthemum crystallinum *and the halophytes *Suaeda *spp., *Salicornia *spp., and *Atriplex *spp. [26,27,40-50].

In this study, the results derived from a large-scale transcriptomic analysis of *A. hypochondriacus *plants performed by massive parallel pyrosequencing technology, are described. The data includes genes found to be specifically- or highly-expressed in stems and also in leaves under four different stress conditions (drought and salt stress, insect herbivory and bacterial infection). This allowed the identification of several stress-responsive genes, including many with unknown function and/or that are expressed in multiple conditions of stress. These may constitute potentially novel mechanisms utilized by this, and related plant species, to deal with highly unfavorable conditions. A comparison of the *A. hypochondriacus *and *A. tuberculatus*, a weedy amaranth species, transcriptomes yielded low levels of similarity. Annotation of homologous transcripts in both species indicated that the majority was associated with genes required for basic biological processes, although an important fraction of them included abiotic stress-related genes.

## Methods

### Sample preparation for 454 sequencing

Seeds of *Amaranthus hypochondriacus *cultivar Revancha and of accession 38040 (origin: India) were kindly provided by E. Espitia (INIFAP, México) and D. Brenner (USDA, Iowa State University, Ames, IA), respectively. Seeds were germinated in 60-well germinating trays filled with a sterile soil preparation composed of a general soil mixture (three parts Sunshine Mix 3TM [SunGro Horticulture, Bellevue, WA], one part loam, two parts mulch, one part vermiculite [SunGro Hort] and one part perlite [Termolita S.A., Nuevo León, México] and coconut paste [Hummert de México, Morelos, México] in a 1:1 v/v relation). The trays were maintained in a growth chamber kept at 26°C, ≈75% R.H. and with a 16: 8 h light (at approximately 300 μmol m^-2 ^s^-1^) dark photoperiod. Amaranth plantlets were subsequently transplanted to 1.3-L plastic pots, containing sterile general soil mixture, 21 days after germination. They were fertilized once, one week after transplant, with a 20:10:20 (N: P: K) nutrient soil drench solution according to the manufacturer's instructions (Peters Professional; Scotts-Sierra Horticultural Products, Marysville, OH, USA). Plants having six expanded leaves were employed for experimentation. Total RNA was obtained from leaves (*A. hypochondriacus *cv. Revancha) or pigmented stems (*A. hypochondriacus *India 38040) using the Trizol reagent (Invitrogen Corp., Carlsbad, CA, USA) as instructed, treated with RNAase-free DNAase and re-purified with the RNeasy kit (Qiagen, Valencia, CA, USA) following the manufacturer's protocol. Different sources of RNA were used to generate the six cDNA libraries employed for pyrosequencing runs: i) leaves of intact plants grown under natural greenhouse conditions in the summer of 2009 (Source 1, S1) ; ii) pooled damaged leaf tissue from plants subjected to herbivory for 1, 4 and 12 h (≈20% maximum leaf-tissue loss) by larvae of the salt marsh caterpillar *Estigmene acrea *(S2); iii ) leaves of noticeably wilted plants resulting from the drought-stress imposed after withholding watering for 3 days (S3) (drought-stress was most probably caused by the confinement of the treated plants in pots, which impeded taproot elongation, a known morphological response to drought in amaranth [see above]), and iv) leaves of plants, showing increased thickness and coarser leaf texture as a result of the acute salt-stress produced by watering the plants for three straight days with 100 ml of a 400 mM NaCl solution, (S4). Leaf material was also obtained from leaves of plants infected with *Pseudomonas argentinensis*, a bacterial amaranth pathogen, as described previously [51] (S5) and from pigmented (red) stem tissue of un-stressed 38040 plants (S6). RNA source S1 to S5 were obtained exclusively from plants of the Revancha cultivar.

### cDNA library construction for pyrosequencing

Two different methods were employed for the generation of the cDNA libraries. In method 1, cDNA synthesis (S1) was performed by using SMART II™ cDNA Synthesis kit (Clontech Laboratories, Inc., Mountain View, CA, USA) following manufacturer's recommendations. The SMART II oligonucleotide (Clontech), which has extra G nucleotides at its 3' end, was used to create an extended template useful for full-length cDNA enrichment. Double stranded cDNA was quantified with a spectrophotometer (Nano Drop 1000, Thermo Scientific, Wilmington, DE, USA) and then concentrated by speed vacuum to a concentration of 500 ng/ul. The products were run on a 2% agarose gel to verify cDNA quality and fragment length. The main size distribution was within the 500 to 4,000 bp range. Approximately 5 μg of each cDNA sample were sheared via nebulization into small fragments, and then sequenced (runs 1 and 2; see below).

In method 2, cDNA synthesis (S2 - S6; destined for the differential gene expression analysis) was performed following a previously described RNA amplification protocol [52]. This procedure is based on a reverse transcription with an oligo(dT) primer bearing a T7 promoter using ArrayScript™ reverse transcriptase (RT), engineered to produce higher yields of first-strand cDNA than wild-type enzymes. ArrayScript RT catalyzes the synthesis of almost exclusively full-length cDNAs. The cDNAs then undergo a second-strand synthesis and cleanup to get a template suitable for *in vitro *transcription with the T7 RNA polymerase. This methodology generates hundreds to thousands of antisense RNA copies of each mRNA in a sample (also called cRNA) from which a second round of cDNA synthesis is performed. This RNA amplification methodology was originally developed as a method to increase very small amounts RNA samples to produce enough material for microarray hybridization [53]. Moreover, several previous reports have confirmed that no bias is generated by the amplification of RNA [54-56].

Steps from aRNA isolation through to pyrosequencing were performed as a service by the National Laboratory of Genomics for Biodiversity (Langebio) at Cinvestav, Irapuato México. Preliminary titration runs were followed by six micro-bead sequencing runs, using Roche-454 GS FLX (454 Life Sciences/Roche; Branford, CT, USA) (runs 1 and 2) and Roche- 454 GS-FLXTM (runs 3 to 6) instruments, respectively. The first two runs involved cDNAs derived from S1. Runs 3 and 4 were done with S2 and S3. The two final runs (5 and 6) involved equimolar cDNA amounts derived from S2, S3, S4 and S5 and S2, S3, S4 and S6, respectively. In runs 5 and 6, the respective cDNAs were placed in defined sections of the pico-titer plate, which was equally divided into four sectors, to permit identification for subsequent analysis (i.e. the digital expression analysis; see below).

### Bioinformatics

The 454-reads were assembled using software version 2.3 Newbler, which has a cDNA option for transcriptome assembly. This option allows the formation of isogroups (a collection of isotigs and/or contigs). In broad terms, isotigs are transcripts, built out of the contigs. Different isotigs within the same isogroup represent alternative splice variants. Thus, an isogroup can be considered the equivalent of a gene.

The resulting sequence set (contigs/isotigs) was annotated using Basic Local Alignment Search Tool (BLASTX) [57] against the non-redundant (nr) database from the National Center for Biotechnology Information (NCBI) (http://www.ncbi.nlm.nih.gov), the Arabidopsis database from The Arabidopsis Information Resource (TAIR) (http://arabidopsis.org/index.jsp), the UniRef50 and UniRef100 databases (UniProt Reference Clusters; European Bioinformatics Institute) and all the Amaranthaceae sequences (ESTs) downloaded from Gen-Bank. Those sequences that did not produce a significant hit (*E *≥ 1 × 10^-10^) with the nr database (3901 sequences; ≈15% of the total) were compared to the PFAM database for annotation. The latter comprises a large collection of multiple sequence alignments and hidden Markov models covering many common protein domains, [58]. Significant BLAST results against TAIR database were used for functional gene ontology (GO) annotation [59].

### Transcriptome comparison: *A. tuberculatus *vs. *A. hypochondriacus*

The raw sequence files (SRR039408, SRR039411 and SRR039412) derived from the recently reported *A. tuberculatus *transcriptome pyrosequencing effort [32] were downloaded directly from the NCBI Sequence Read Archive (SRA) at http://trace.ncbi.nlm.nih.gov/Traces/sra/sra.cgi?study=SRP002251. Reads were assembled after quality control, following an identical procedure as that used for *A. hypochondriacus*. Transcript annotation for *A. tuberculatus *was performed by querying the UniRef 100, and Amaranthaceae ESTs databases. Both transcriptomes were then aligned with each other using BLASTN to identify homologous contigs. Sequence homology was defined only at *E *values ≤ 1 × 10^-10 ^and identity ≥ 90%. Homologous transcripts were quantified and classified into five different categories, i.e. those: i) producing the same hit; ii) different hits; iii) and iv) one hit for one species and no hit for the other, and vice-versa, or v) no hit, when queried against the above databases. Annotated transcripts detected only in *A. hypochondriacus *or *A. tuberculatus *were also quantified.

### Digital expression analysis

The number of reads per gene was counted in each of the 454-sequencing outputs derived from the salt stress, water stress, insect herbivory and bacterial infection treatments and also from stem tissue (runs 5 and 6). Genes having read counts lower than 5 were eliminated. To calculate relative expression profiles in each stress treatment, Relative Abundance (RA) values were computed for each gene per treatment sample by dividing its 454-sequence count by the total 454-sequence count in the treatment sample. Differentially expressed genes in one or more treatments were detected by using the R [60] and χ^2 ^test statistics using a freely available web tool (http://telethon.bio.unipd.it/bioinfo/IDEG6_form/) [61]. A gene was considered to be differentially expressed when at least one statistical test yielded significance values ≤ 0.0001. A similar procedure was employed to identify transcripts that were stem-specific or highly abundant in this tissue.

The following considerations were adopted for the organization of the digital stress-related gene expression data: i) a *minimum *(MIN) or baseline/control expression value for a given gene was assigned to the lowest RA in the four-treatment set examined. The RAs that produced an expression ratio ≤ 2 when divided by MIN were also considered as MINs; ii) a gene was considered to be *significantly expressed *(SE) by a given treatment when its RA yielded a ratio ≥ 2 when divided by MIN, and iii) *maximum expression *(ME) levels for a given gene were assigned to the treatment having the highest SE. Treatments were reported to produce additional MEs when their respective SEs yielded a ratio ≤ 2 when divided by ME. This classification was devised to give an indication of the influence that a given treatment or group of treatments had on the expression levels of a particular gene. Six basic patterns of expression could be generated on the basis of the above definitions: 1) induced expression in only one treatment (only MEs); 2) induced expression in two treatments (ME-ME or ME-SE combinations) and 3) induced expression in three treatments (ME-ME-ME, ME-ME-SE or ME-SE-SE combinations]). A total of 50 different patterns of expression were produced when all four stress treatments analyzed in this study were accommodated into the above basic patterns.

## Results and Discussion

### Roche GS-FLX and GS-FLXTM sequencing and assembly

**Figure 1:**
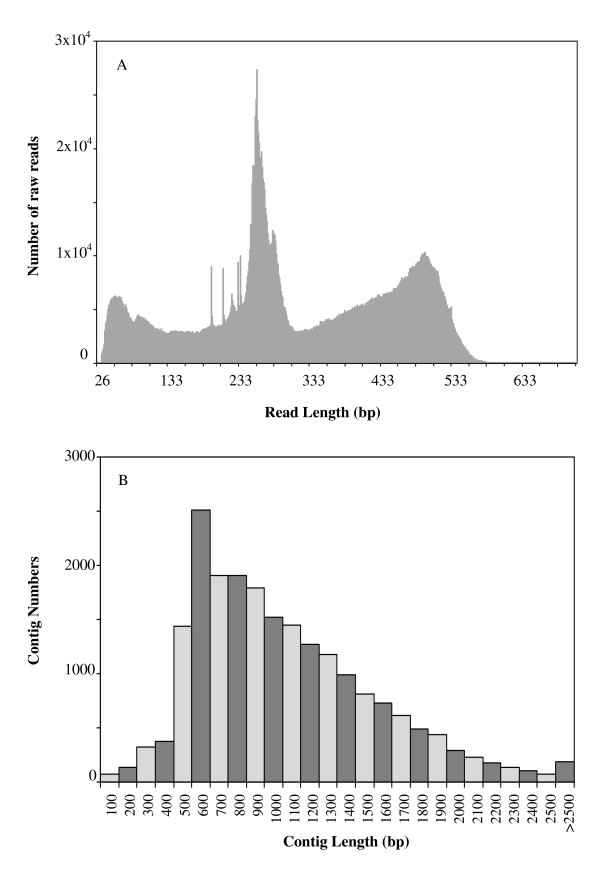
**Length frequency distribution of *Amaranthus hypochondriacus *raw reads (A) and assembled isotigs/contigs (B)**. Reads distribution reflects the utilization of different pyrosequencing platforms (GS-FLX 454 and GS-FLXTM).

Six sequencing runs yielded ≈910 Mb total data size equivalent to 2,913,966 raw reads. The raw sequence files are available from the NCBI Sequence Read Archive (SRA) at http://trace.ncbi.nlm.nih.gov/Traces/sra/sra.cgi?study=SRP006173, as files SRR172675 (S1), SRR172676 and SRR183482 (S2), SRR172677 (S3), SRR172678 and SRR183483 (S4), SRR172679 (S5) and SRR172680 (S6). Length frequency distribution of raw reads clustered around the 200-to-300 bp and 300-to-400 bp range as the result of using two different platforms for sequencing (Figure 1A). A total of 2,700,168 reads (93% of total) entered into the assembly process which yielded 21,207 high quality assembled sequences (20,408 isotigs + 799 contigs¸ equivalent to 87% of reads entering assembly and ≈82% of all assembled sequences). These ranged in length from 80 to 3,379 bp (Figure 1B) and had an average sequence length of 1,014 bp (isotigs) and 930 bp (contigs). A total of 178,636 reads (≈6% of total) remained as singletons (coverage depth = 1); of these, only 5,113 clean sequences remained after quality control. Isotigs were further incorporated into 15,667 isogroups. A status summary of the sequencing, assembly and annotation (see below) process is presented in Table 1.

**Table 1:** Summary of *A. hypochondriacus *454 sequencing data trimming, assembly and annotation

Run Metrics	Total raw reads	2,913,966 (100%)
	Total bases	909,631,600
	Reads after quality control and trimming	2,700,168 (92.6%)
	Bases entering assembly	877,153,000 (96.4%)
**Assembly**	Aligned reads	2,417,008 (89.5%)
	Aligned bases	803,229,499 (88.3%)
	Assembled reads	1,886,081
	Fully assembled	1,422,449
	Partially assembled	463,632
	Singletons (5.9)	178,636
	Repeats	68,980
	Outliers	56,216
	Too short	46,623

**Isogroup Metrics**	Total isogroups	15,667
	Average contig content	3.0
	Largest contig content	22,172
	Number with one contig	12,739
	Average isotig content	1.3
	Largest isotig content	52
	Number with one isotig	12,950

**Isotig metrics**	Total isotigs	20,408
	Average contig content	1.7
	Largest contig content	17
	Number with one contig	12,985
	Number of bases	20,710, 069
	Average isotig size	1, 014
	N50 isotig size	1,196
	Largest isotig size	4,762

**Large contig metrics**	Number of contigs	15,608
	Number of bases	15,170,717
	Average contig size	971
	N50 contig size	1,063
	Largest contig size	3,379

**All contig metrics**	Number of contigs	25,998
	Number of bases	18,043,010

**Annotation (contigs/isotigs)**	nr (NCBI)	17,282
	TAIR	16,597

**Annotation (singletons)**	UniRef 100	17,440
	UniRef 50	4,396
	Amaranthaceae ESTs	10,846
	TAIR	≈1,000

### Annotation of *A. hypochondriacus *contigs/isotigs

All contigs/isotigs were queried against the nr, TAIR, UniRef100, UniRef50 and Amaranthaceae ESTs and PFAM databases for annotation. Approximately 82% of all entries produced significant hits (*E *≤ 1 × 10^-10^) when queried against the nr database (Table 1). The 3,901 sequences with no significant hit versus the nr database were queried against the PFAM protein domain database in order to determine their putative function. Only a small fraction of these sequences (≈2%) produced significant hits (*E *values ≤ 1 × 10^-5^) to known protein domains. These results are available in Additional file 1. Annotation of the 5,113 clean singletons against the TAIR database yielded approximately 1,000 significant hits.

**Figure 2:**
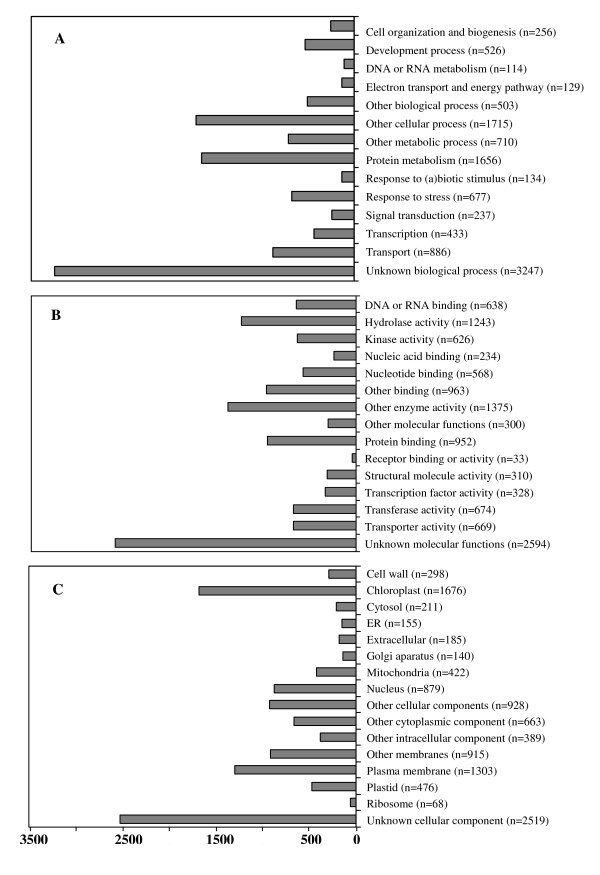
**Summary of *Gene Ontology *functional annotation of GS-FLX 454 and GS FLXTM isotigs/contigs**. Annotated sequences (vs TAIR database) were classified into (**A**) 'Biological Process', (**B**) 'Molecular Function' and (**C**) 'Cellular Component' groups and 45 subgroups.

**Figure 3:**
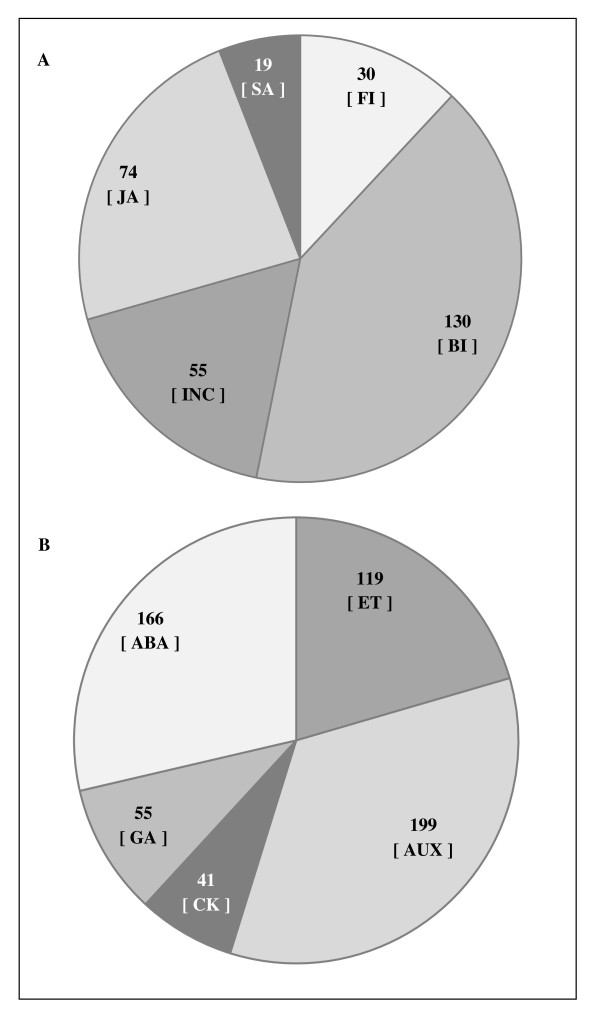
**Number of *A. hypochondriacus *isotigs/contigs categorized within the Response to Biotic Stress (A) and phytohormone function (B)**. BI, FI, INC, JA and SA represent bacterial and fungal infection, incompatible plant-pathogen interaction, jasmonic acid and salicylic acid, respectively. ABA, AUX, CK, ET and GA represent abscisic acid, auxins, cytokinins, ethylene and gibberellins, respectively.

The best hit for each unigene queried against the TAIR database was utilized to assign functional GO annotation in terms of *biological process *(11,224 sequences), *molecular function *(11,499 sequences) and *cellular component *(11,227 sequences) *groups*. The results are summarized in Figure 2. As expected, the largest percentage in each GO group (12% to 15%) was conformed by contigs/isotigs with an unknown functional annotation. No obvious differences in the number of sequences assigned to each category, including response to (a)biotic stress, were observed between grain amaranth and *Arabidopsis thaliana*. This was probably a reflection of Arabidopsis' known capacity respond strongly to abiotic and biotic stresses at the transcriptional level [62,63]. This outcome also argues against the possibility of grain amaranth possessing a different transcriptomic signature, particularly in the stress and response to stimuli categories, that could explain its characteristic (a)biotic stress tolerance, in contrast to what has been observed in plant species adapted to extreme habitats (e.g. the Arabidopsis-related halophyte *Thellungiella halophila *[64] and extremophile mangroves [65]). Thus, functional GO assignment for *Biological Process *(Figure 2A) indicated that 3% of the contigs/isotigs were grouped under stress/stimuli response, 2% in development processes and an additional 4% in other biological and metabolic processes. These categories were of our particular interest considering that one of the primal objectives of this transcriptome study was to provide information leading to the identification of (a)biotic stress-responsive genes (see below). From the number of transcripts to which a defense role was assigned (1% of total), more than half were associated with bacterial infection (41%) and jasmonic acid (JA)-regulation (24%), including many JA biosynthetic (e.g. *LOX13*, *AOS, AOC, OPR3*) and JA-responsive genes (Figure 3A; see also additional files 2, 3 and 4).

The overall perspective obtained from the above information is that grain amaranth possesses a diverse arsenal of genes to resist pathogen infection and insect herbivory, the majority of which are reported for the first time in this species. These include genes potentially involved in oxalate and phytoecdysteroid synthesis (results not shown), which are believed to be effective defensive weapons in amaranth and other species [66-68]. The implementation of a relatively robust defense response was somewhat unexpected, at least against insect herbivory, considering that the unusually high tolerance to defoliation we have observed in *A. hypochondriacus *plants (see below), might be expected to exempt this species from an investment in metabolically costly inducible defense responses (e.g. protease inhibitors and lectins). The nature of the pathogen-resistant genes isolated was also complex, and included a whole gamut of bacterial and fungal elicitor-induced and pathogenesis-related proteins, extracellular receptors similar to those involved in elicitor-induced defense responses, proteases, transcription factors (TFs) and enzymes involved in reactive oxygen species generation-detoxification.

Also important from our perspective were genes potentially involved in compensatory photosynthesis, carbohydrate re-localization (Table 2) and regulation/synthesis of phytohormone levels (Figure 3B), possibly related to the increased ramification observed in grain amaranth plants as a response to defoliation caused by insect herbivory and/or mechanical damage [40,69]. Many of the genes identified can be used for studying unrelated processes. For example, the analysis of phytohormone-related genes, in combination with those showing homology with flowering genes is being pursued to gain an insight of the genetic mechanisms responsible for the several symptoms produced by phytoplasm infection of grain amaranth in the field, including phyllody [70].

**Table 2:** Selected genes related to carbohydrate (CHO) synthesis metabolism, storage and mobilization

Gene description	No. Isotigs
Starch synthase I	3
Starch synthase II	7
Starch synthase III	1
Starch synthase V	3
Starch synthase VI	1
Granule-bound starch synthase I	2
Starch ramifying enzyme I	5
Starch ramifying enzyme II	1
Starch phosphorylase I	1
Starch phosphorylase H	4
Pullulanase	1
Iso-amylase II	2
Iso-amylase III	1
SnRK1 (SNF1-Related Protein Kinase-1)	2
SNF4 (Sucrose non-fermenting-4)	1
Glucose-6-P/phosphate transporter	5
Phosphoenol pyruvate/phosphate transporter	8
Triose P/phosphate transporter	8
AGPase small subunit	1
AGPase large subunit	3
Sucrose synthase	7
Invertase (vacuolar)	4
Invertase (neutral/alkaline)	14
Invertase (cell wall)	1
Invertase inhibitors/PMEI	7
P-glucomutase	10

The genes listed were identified in the GS-FLX 454 and GS-FLXTM pyrosequencing of *A. hypochondriacus *and could be potentially involved in CHO re-localization associated with tolerance to defoliation by insect herbivory or mechanical damage.

### Transcriptome comparison between *A. hypochondriacus *and *A. tuberculatus*

The publicly available raw transcriptomic 454 pyro-sequencing data generated for *A. tuberculatus *[32] was re-assembled using the same computational methods as for *A. hypochondriacus*. In our hands, however, the assembly yielded a ratio of contigs/singletons (12,216/53,803) that differed from the one reported by the former workers (22,035/22,434), perhaps as a consequence of the use of different assemblers [71]. The discrepancy occurred despite the fact that 83% of the total *A. tuberculatus *raw reads entering the process was assembled. BLASTN alignment of the resulting 12,216 *A. tuberculatus *contigs with the 21,207 *A. hypochondriacus *isotigs/contigs yielded 8,260 homologous sequences (*E *≤ 1 × 10^-10 ^and ≥ 90% identity). The number of contigs from each species that produced significant hits (E ≤ 1 × 10^-10^) when queried against the Uniref 100 and Amaranthaceae ESTs data bases, is shown in Table 3. Combined use of above information led to the quantification of the number of homologous contigs producing the same hit, different hits, one hit for one species and none for the other, and vice-versa, and no hit. The results obtained are shown in Table 4. The analysis of the homologous transcripts annotated with the Amaranthaceae EST data base indicated that the majority had an unknown function/provenance (21%). The highest proportion (71%) was found in EST libraries generated from immature seed and floral tissues in *Chenopodium quinoa *[72], inflorescence, germinating tissue, roots in various stages of development, hypocotyls, seed stalks and cotyledons of beet root and chlorenchyma cells of the non-Kranz C4 species *Bienertia sinuspersici *[73]. Stress related genes constituted the smallest fraction (8%), mostly represented by ESTs generated from salt-stress halophyte species (*Salicornia brachiata *[44], *Suaeda salsa *[74], *S. maritima *[46], *Atriplex centralasiatica *[75] and *C. glaucum*, in addition to ESTs from immature tissue of *Salsola tragus*. All the biotic-stress related transcripts identified came from cDNA libraries of beet roots subjected to maggot (*Tetanops myopaeformis*) feeding [76,77]. On the other hand, two thirds of the homologous transcripts annotated with the Uniref100 data base had an unknown function. Subsequent classification of transcripts (33%) having an assigned function in the biological processes category placed the majority of them (16%) within a group consisting of basic house-keeping functions (e.g. cellular component organization and biogenesis, cell cycle, cell death, regulation of gene expression, translation, cellular homeostasis, anatomical structure morphogenesis and growth, carbohydrate, protein and DNA metabolic processes, transport and photosynthesis), primary and secondary metabolism (7%), signal transduction and transcription regulation (4%). The rest included transcripts expressed in response to biotic (2%) and abiotic stress (4%). The majority of the latter were isolated from Amaranthaceae and related halophytes mostly exposed to salt stress, Interesting (a)biotic stress-related genes present in both species include a plastid-lipid associated protein known to be induced in response to multiple stresses in many plant species [78], AtPOB1, a BTB/POZ-domain protein that was found the to positively regulate disease responses in Arabidopsis and tobacco [79], the phloem sap protein AtPP2-A1 whose over-expression in Arabidopsis strongly repressed phloem feeding of the green peach aphid *Myzus persicae *[80], a transcript similar to the non-specific lipid-transfer protein type 2 from *Tamarix hispida*, whose expression was found to be part of an adaptive response to abiotic stresses in this species [81], polyamine oxidase, an H_2_O_2 _producing enzyme supposedly involved in cell wall differentiation processes and defense responses, which was recently found to be required for wound healing in maize [82], methionine sulfoxide reductase, found to be active in defense against pathogens in pepper plants, via the regulation of cell redox status [83], and the DEAD-box ATP-dependent RNA helicase 7, a type of DNA repair protein recently shown to confer multi-stress resistance when expressed in plants [84-86]. Also remarkable was the identification several genes related to heavy metal ion homeostasis and tolerance, cation detoxification, water transport and stress-related phytohormone (e.g. abscisic acid and JA) biosynthesis and signal transduction (see additional file 5).

**Table 3:** Comparison of *A. hypochondriacus *(*Ah*) and *A. tuberculatus *(*At*) transcriptomes (I)

Species	UniRef100	Amaranthaceae ESTs
*A. hypochondriacus*	17,440	10,846
*A. tuberculatus*	6,625	7,185

Number of sequences with significant hits (*E *≤ 1 × 10^-10^) to the UniRef 100 and Amaranthaceae ESTs databases in each species.

**Table 4:** Comparison of *A. hypochondriacus *(*Ah*) and *A. tuberculatus *(*At*) transcriptomes (II). Annotation of homologous contigs

Annotation	UniRef100	Amaranthaceae ESTs
Homologous contigs with different hit	1,406	2,394
Homologous contigs with same hit	2,858	2,331
Homologous contigs with no hit	559	1,088
*Ah *contig with hit but not its *At *homologue	1,406	757
*At *contig with hit but not its *Ah *homologue	235	1,690

The number of annotated transcripts that were detected in only one species was comparatively large (Table 5). An illustrative example of the differences observed between weedy and grain amaranth transcriptomes is given by the analysis of herbicide-target genes that were annotated with the UniRef 100 and Amaranthaceae ESTs databases. It indicated that 29 of these were found in both species, whereas 13 and 8 sequences were found only in *A. hypochondriacus *and *A. tuberculatus*, respectively (Table 6).

**Table 5:** Comparison of *A. hypochondriacus *(*Ah*) and *A. tuberculatus *(*At*) transcriptomes (III)

Species	UniRef100	Amaranthaceae ESTs
*A. hypochondriacus*	9,974	5,364
*A. tuberculatus*	2,222	2,750

Number of annotated transcripts detected exclusively in one species.

**Table 6:** Comparison of *A. hypochondriacus *(*Ah*) and *A. tuberculatus *(*At*) transcriptomes: number of hits (isotigs/contigs) to herbicide target-site genes in the UniRef 100 and other databases

	UniRef 100 Annotation			**Annotation: all databases**^**a**^
Herbicide Target-site Gene	Hit in *Ah *and *At*	Hit in *Ah *only	Hit in *At *only	*Ah*
Tubulin	11	4	4	34
Acetolactate synthase	2	0	1	5
Protoporphyrinogen oxidase	1	1	2	2
Glutamine synthetase	6	1	0	11
1-Deoxy-D-Xylulose-5-phosphate synthase	3	3	1	3
4-Hydroxyphenylpyruvate dioxygenase	3	0	0	3
Acetyl-CoA carboxylase	1	4	0	13
Phytoene desaturase	2	0	0	2
5-Enolpyruvylshikimate-3-phosphate synthase	0	0	0	1
Dihydropteroate synthase	0	0	0	1
D1 protein (plastidic gene)	0	0	0	2

^a^nr, TAIR, UniRef 100, UniRef 50, Amaranthaceae ESTs databases.

The rather stringent parameters employed for the transcriptome comparison could have led to the transcriptome differences herein observed, although the use of lower E-value thresholds (say *E *≤ 1 × 10^-5^) might have not contributed much to increase level of transcript homology, as suggested by a previous genome sequencing study in *Eucalyptus grandis *[87]. However, another more plausible possible explanation is that the above discrepancies were the reflection of fundamental differences in the overall experimental design utilized to generate both transcriptomic data. For instance, many biotic stress-related genes detected in *A. hypochondriacus *were absent in A. *tuberculatus *(results not shown). An alternative hypotheses proposing that the difference observed was due to an important sequence divergence occurred during speciation/domestication will require much further research to be validated.

### Digital expression profiling

#### Stress-responsive transcriptional profile in leaves

**Figure 4:**
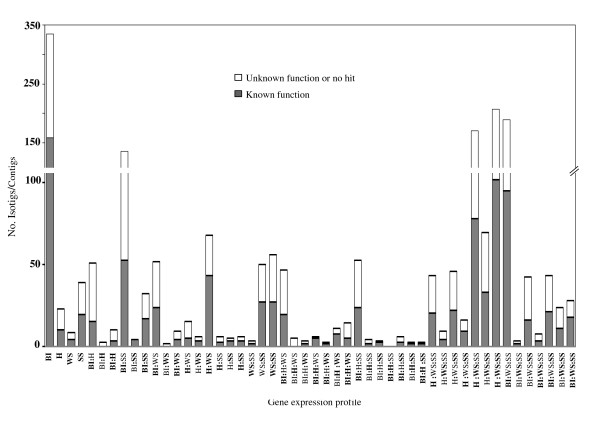
**Number of contigs/isotigs within the 50 gene expression combinations generated to categorize digital expression data**. Number of significantly expressed genes in response to: salt stress [SS], water stress [WS], insect herbivory [H] and bacterial infection [BI]). Bold letters represent *Maximum Expression *values (see text).

This technique, also known as tag sampling or RNA-seq, is considered to be an efficient method for gene expression analysis [88,89]. The digital expression profiling analysis performed for *A. hypochondriacus *identified a total of 1,971 differentially expressed genes in response to at least one of the four stress treatments tested (i.e. water stress, salt stress, insect herbivory and bacterial infection) (Additional file 6). Fifty different gene expression profiles were generated to determine the influence of any given stress treatment on the expression levels of a particular gene. The results are shown in Figure 4. An evident feature of this analysis was the high percentage of un-annotated genes or genes with unknown function that were induced by stress. These represent a potentially rich source of genetic material that could be systematically analyzed for the discovery of genes involved in novel mechanisms of stress resistance.

All the stress-inducible genes with known function that were identified in 41 of the 50 gene expression categories were also tabulated (Additional file 7). These included several TFs known to be regulators of stress responses in other plant species, e.g. AREB-like protein [90], Dof-type zinc finger domain-containing protein [91], BTB/POZ domain-containing protein [92], GRF zinc finger containing protein [93], RAP 2.4-like protein [94], JAZ1 repressor [95], ATEBP/ERF72/RAP2.3 (related to AP2-3) [96], RAV [97], MYB-like transcription factor [98], TINY-like protein 2 [99], Cys2/His2 zinc-finger transcription factor [100], the little known GAGA-motif binding transcriptional activator [101]; SCOF-1 zinc finger proteins, found to be induced by cold or salt stress in Arabidopsis and other plants, apparently to enhance ABRE-dependent gene expression [102], a putative NAC transcription factor [103], and histone-fold/TFIID-TAF/NF-Y [104]. Others have been identified in several xerophytes/halophytes as possible factors that contribute to their ability to colonize extreme habitats, e.g. lycopene synthase [105] water channel proteins [106], myo-inositol-1-phosphate synthase, [107] cystathionine gamma-synthase [108] phosphoenolpyruvate carboxylase [109], Na^+^/H^+ ^antiporter [110], protein phosphatase-2C [111], Ca^2+^/H^+ ^antiporter [112], calcineurin B-like protein [113], inositol monophosphatase [46], and salt-induced hydrophilic protein [114].

Not surprisingly, numerous transcripts coding for reactive oxygen scavengers were found to be strongly induced, many of them by multiple stresses, e.g. [Fe] superoxide dismutase, glutathione S-transferase Z1, germin-like oxidase and several catalases, peroxidases and ascorbate peroxidases. Also, the strong and multiple-stress induction of aspartyl protease, various cysteine proteases, a subtilisin-like protease and a vacuolar processing enzyme (VPE) supports a role for protein-recycling processes in response to stress, similarly to what was found during the salinity stress adaptation competence process in the extremophile *T. halophila *[115], whereas the expression of expansins, xyloglucan endotransglycosylases, several cellulose synthase subunits, glycine-, proline- and hydroxyproline-rich proteins is supported by the observed capacity to adjust cell wall properties in many plants undergoing stress [116,117]. Many of these carbohydrate-active genes were also highly expressed in stems (see below).

Of particular importance were genes highly expressed by several stress treatments, not previously reported in amaranth or related halophyles/extremophyles. These have obvious potential biotechnological applications and could also contribute to the elucidation of molecular mechanisms leading to resistance to multiple stress conditions. A selection includes the following: *Drm3*, required for *de novo *DNA methylation in *Arabidopsis thaliana *where it is proposed to regulate gene silencing processes [118]; *Enhancer of SOS 3-*1 which encodes a chloroplast-localized protein that interacts with the critical SOS3 and SOS2 regulators of salt stress tolerance in *Arabidopsis *[119,120]; YCF3 and HCF101 (high chlorophyll fluorescence 101) proteins deemed to be essential for assembly and accumulation of the photosystem I (PSI) complex and prevention of photo-oxidative damage [121,122]; translational initiation factor eIF1, found to be a determinant of sodium tolerance in yeast and plants, implying that translation is a salt toxicity target and that its recovery might be a crucial mechanism for cell survival under NaCl stress conditions [123] in addition to its proposed regulation of ion accumulation and the intracellular redox status [124]; ATP-dependent FtsH protease 9, involved in the degradation of the D1 protein of photo-damaged (PSII), a step which is needed to avoid the accumulation of excessive levels of reactive oxygen species [125]; the ACD1-LIKE electron carrier, resembling the *Arabidopsis-accelerated cell death *gene product, involved in the oxygenation of pheophorbide *a *that is required to prevent photooxidative destruction of the cell and also found to be up-regulated during salt stress adaptation process in *T*. *halophila *[115,126]; the prohibitin gene *PHB1*, family members of which have been found to accumulate in response to different stress conditions in many plants, presumably to act as safeguards of mitochondrial function and integrity, triggers for the retrograde mitochondrion-to-nucleus signaling and/or mediators of the interplay between H_2_O_2 _and NO, by a still undefined mechanism [127]; the Yellow Stripe Like 6 protein, whose members are hypothesized to participate in the delivery of metal micronutrients to and from vascular tissues and in metal tolerance and hyper-accumulation [128]; putative linker histone H1 variant protein, expressed by drought stress conditions in tomato, and acting by a mechanism other than chromatin organization that is proposed to involve a negative regulation of stomatal conductance [129]; GASA-1/LtCOR1-like, a gibberellin regulated protein putatively involved in the regulation of fruit ripening [130] or the establishment of the dormant state in cambial meristems of trees [131]; beta and gamma-tubulin chains, whose expression is coincident with the increasingly important role played by the cytoskeleton in the mediation of the plant cell's response to stress [132,133]; translation initiation factor 5A, found to be involved in an apparently isoform-dependent regulation of stress response pathways and resistance through a largely unknown mechanism [134]; *argonaute 4-like *gene, the primary protein involved in methylation of heterochromatin and recently recognized as a critical factor for small RNA mediated systemic signaling required for plant (a)biotic stress responses and nutrient deprivation [135,136]; a putative arginase, highlighting the role of arginine as a precursor for the biosynthesis of polyamines and nitric oxide, employed as messengers for the adaptation of plants to stress [137-139], and pore-forming toxin-like lectin protein Hfr-2, recognized as an important biotic resistance factor in wheat against Hessian fly infestation and fungal (*Puccinia striiformis*) infection, and implicated in the vegetative phase change in maize [140-143], but with no known function in abiotic stress regulation. The functional characterization of a select set of multi-stress-inducible *A. hypochondriacus *genes, in Arabidopsis, tobacco and/or grain amaranth, is now under progress in our laboratory.

#### Transcriptional profile in stems

Comparison of the stem-derived cDNA library (S6) with those generated from leaves subjected to biotic and abiotic stress (S2 to S5) permitted to identify a small group of transcripts whose expression was exclusively detected in stems. Remarkably, the accumulation of several other transcripts was higher in stems than in foliar tissue of amaranth plants exposed to (a)biotic stress (see additional file 8). The transcript profile observed was consistent with previously data reported for stem transcriptomic analyses in *Arabidopsis thaliana *[144,145]. All annotated transcripts were classified into different categories, similarly to the above studies.

Lignin and cuticule wax biosynthesis was represented by genes coding for proteins presumably involved in monolignol biosynthesis (e.g. cytochrome P450 reductases, needed for the activity of several key cytochrome P450 enzymes of the phenylpropanoid pathway [144]), monolignol transport (e.g. ABC transporters [146]) and cuticular lipid export (e.g. white-brown-complex ABC transporter family [147]). The modest number of up-regulated lignin biosynthesis genes that were detected was probably related to the use of young amaranth plants, not yet undergoing active lignification, for experimentation.

The carbohydrate-active enzyme category was highly represented. This was not surprising considering that these proteins play a fundamental role in cell wall biosynthesis and modification and are therefore tightly regulated during stem development. It included a number of glycosyl transferases and several glycosyl hydrolases (GH) representing families having cellulase (GH9), β-1,3-glucanase (GH3), xylanase (GH10), xyloglucan endotransglucosylase-hydrolase (GH16), glucan endo-1,3-beta-D-glucosidase (GH17), invertase (GH31) and β-D-galactosidase (GH35) activity. These enzymes are variously required for cell wall loosening and elongation, formation of the secondary cell walls of vascular tissues, hydrolysis of the xylan backbone, post-translational modifications (as glycosylations) of proteins and mobilization of energy in form of sucrose. Also detected were pectin methylesterases (PME) involved in the modification of the physical, chemical, and biological properties of pectins. The concomitant expression of a PME inhibitor probably represented a need to regulate PME in young amaranth stems in order to avoid the wall rigidification associated with PME activity. In addition, a putative β-expansin protein was detected; these proteins modulate the interaction between hemi-celluloses and cellulose presumably via a disruption of their shared hydrogen bonds [145].

Within the extracellular oxido-reductases group were found two peroxidases, belonging to the peroxidase 25 and 64 families, respectively. Peroxidases have been found to be expressed at moderate to high levels in developing stems, where they are believed to reduce cell wall extensibility due to their role in the formation of covalent links between pectin residues, hydroxyproline-rich proteins like extensins, and lignin precursors. One gene encoding a multicopper oxidase of the SKS family (*SKS5*) was identified. The function of these proteins in stem development is not well known, although the expression of *SKS5 *was latterly found to be up-regulated in metal hyper-accumulating ecotypes of *Thlaspi caerulescens *[148]. Another oxido-reductase identified in amaranth stems was an 2-OG-Fe(II) oxygenase protein of unknown function that was recently found to be associated with defense mechanisms against fungal infection in Arabidopsis [149].

Several genes encoding proteins with putative interaction domains with polysaccharides and/or other proteins were identified. Many of the genes classified within this category are kinases, peptide receptors and receptor-like kinases that regulate developmental processes in plants such as the CLAVATA1-like receptor [150], CLAVATA3/ESR-related receptor [151], Abnormal Leaf Shape 2 receptor-like kinase [152], leucine-rich repeat receptor-like kinase RLK7 [153] and LRR XI-23 kinase [154]. A number of hydroxiproline-rich (glyco) proteins, most probably representing arabinogalactan-proteins (AGPs), structural proteins (e.g. extensins, proline-rich proteins, PRPs) and a related prolyl 4-hydroxylase (catalytic alpha-2 subunit) needed for the hydroxylation of proline residues [155], were also highly expressed in stems. Numerous roles for AGPs in plant development have been suggested by means of their influence on cell fate determination, somatic embryogenesis, and cell proliferation. Also, AGPs have been assumed to be signal molecules and to associate with pectic polysaccharides, whereas extensins, PRPs and others (e.g. glycine-rich proteins) have been shown to be expressed in specific cell types including xylem and phloem tissues [145].

Also present were genes coding for a Rhomboid-like 2 endopeptidase, and two proteins with inhibitor activity: a lipid transfer protein/trypsin-alpha amylase inhibitor and a cysteine proteinase inhibitor. In addition, transcripts for an F-Box protein (SKIP2) and a 26S proteasome non-ATPase regulatory subunit, known to be involved in the targeted degradation of proteins triggered in response to various stimuli during growth and/or diverse stress conditions, were also detected. It has been suggested that proteinase activity and its modulation by proteinase inhibitors is necessary for the processing and/or turnover of cell wall proteins, generation of peptide signals, programmed cell death and/or balancing cell expansion/proliferation rates, which are collectively required for proper stem development [156,157].

Among the miscellaneous protein category were found genes coding for proteins involved in lipid metabolism (GDSL-lipases [158] and a putative glycerophosphoryl diester phosphodiesterase [159]), which are suggested to be important for stem development, a copper-binding plantacyanin (ARPN), assumed to regulate oxido-reduction processes in cell walls, several proteins known to be required for stem cell maintenance in the shoot apical meristem (histone H2A; [160]; Aurora 2 histone kinase [161]), metal tolerance (e.g. selenocysteine methyltransferase [162]) and components of the cytoskeleton, most probably involved in cell division and elongation [163]. The finding of a transcript coding for the catalytic LigB subunit of an aromatic ring-opening dioxygenase family (i.e. a putative dopa dioxygenase) the prominent enzyme in betacyanin biosynthesis, and of biosynthetically related glycosyl transferases (GTs) (e.g. GT from *Phytolacca Americana *and a UDP-GT) [164] was consistent with the highly pigmented phenotype of the stem tissue used to generate the sequenced cDNA library. The determination of the structure and regulation of pigment-related genes, their tissue- and stress-related expression patterns, and their probable role in defense against insect herbivory in grain amaranth is now being actively pursued in our laboratory. Several TFs were also detected. In accordance with a previous report [144], most of TFs found to be highly expressed in stem tissue of grain amaranth were of the MYB, AP2-EREBP, GRAS, bHLH-domain and homeodomain families (e.g. WOX4 [165]). TFs in stems have been variously associated with the regulation of vascular tissue bio-genesis and differentiation, phenylpropanoid gene expression and fiber development [144]. Finally, a high level of expression was found for several abiotic stress and defense-related genes in stems of *A. hypochondriacus*. The presence of highly expressed defense-related genes was in accordance with a recent report showing that genes involved in plant defense and protective functions were dominant in developing stems of *Populus trichocarpa *[156]. In this respect, the concomitant presence of a putative jasmonate o-methyl transferase and a CXE carboxylesterase gene coding for a protein that can presumably identify methyl jasmonate (MeJA) as its substrate (in addition to methyl salicylate and indol-3-acetate) in *Actinidia arguta*, argues in favor of a possible role for MeJA in signaling, both within and between amaranth plants, during biotic and/or abiotic stress [166,167]. Other interesting genes identified in amaranth stems to which an active role in pathogen defense has been recently ascribed include those coding for an epoxide hydrolase 2 [168] and a VPE-1B [169], respectively. The role of epoxide hydrolase in defense is thought to be associated with its involvement in detoxification, signaling, and/or metabolism of antimicrobial compounds, whereas VPE's importance is believed to derive from its involvement in elicitor-triggered immunity connected with the combined induction of a hypersensitive response (HR) and stomatal closure. As mentioned above, VPE expression has also been associated with responses to abiotic stress.

## Conclusions

The work herewith presented describes the first large-scale 454 pyrosequencing transcriptomic analysis of *A. hypochondriacus*, an under-utilized and stress-tolerant crop known to produce highly nutritious seeds and foliage. This study allowed the identification of numerous genes that are presently been analyzed to determine their role in unknown or poorly understood aspects of grain amaranth physiology, such as the mechanisms employed to tolerate defoliation, either by mechanical damage or insect defoliation. Furthermore, a digital expression analysis of transcriptome-derived data allowed the identification of numerous genes that are expressed in response to (a)biotic stress and also in a stem-specific manner. This information greatly complemented the relatively scant knowledge regarding stress-related gene expression in grain amaranth, particularly with regards to insect herbivory and bacterial infection. Furthermore, it uncovered many multiple-stress genes that could contribute to the effective response capacity against several types of environmental insults often reported in grain amaranth. Finally, a comparison with transcriptomic data obtained from an amaranth weedy species produced large differences in the number and types of transcripts detected. Although this outcome most probably resulted from fundamental experimental differences in the way the respective transcriptomic data was obtained, it is tempting to speculate that such a difference reflected a large degree of divergence between wild and cultivated amaranths generated during speciation and/or as a consequence of the domestication of *A. hypochondriacus*.

## Authors' contributions

JPDF and LHE drafted the manuscript. MGE performed the digital expression analysis. MGE performed the statistical analysis of the digital expression data. JPDF analyzed the transcriptional and digital expression data. HAA and NAMG performed the GO functional annotation. GCA and FPC analyzed the raw transcriptomic and assembly data. KCC, GCA and PACA performed the plant stress treatments. KCC, GCA, PACA, JMS, FPC and EVO analyzed the transcriptomic data pertaining biotic stress, phytohormones and carbohydrate metabolism, as possibly related to tolerance to defoliation by herbivory or mechanical damage. JPDF designed and coordinated the study. All authors read and approved the final manuscript.

## Supplementary Material

Additional file 1 **Functional annotation of clean GS-FLX 454 and GS FLXTM contigs/isotigs by comparison to the PFAM database**. Click here for file

Additional file 2 **Transcripts associated with jasmonic acid-related responses identified in *A. hypochondriacus***. Click here for file

Additional file 3 **Transcripts associated with responses to bacterial infection identified in *A. hypochondriacus***. Click here for file

Additional file 4 **Transcripts associated with the incompatible plant-pathogen interaction identified in *A. hypochondriacus***. Click here for file

Additional file 5 **Annotated (Uniref 100) homologous transcripts in *A. hypochondriacus *and *A. tuberculatus***. Click here for file

Additional file 6 **Digital expression data; total contigs/isotigs that were differentially expressed in response to (a)biotic stress**. Click here for file

Additional file 7 **Digital expression data; total contigs/isotigs having a known function that were differentially expressed in response to (a)biotic stress**. Click here for file

Additional file 8 **Digital expression data; total contigs/isotigs having a known function that were differentially expressed in stems**. Click here for file
